# Efficient linear phase contrast in scanning transmission electron microscopy with matched illumination and detector interferometry

**DOI:** 10.1038/ncomms10719

**Published:** 2016-02-29

**Authors:** Colin Ophus, Jim Ciston, Jordan Pierce, Tyler R. Harvey, Jordan Chess, Benjamin J. McMorran, Cory Czarnik, Harald H. Rose, Peter Ercius

**Affiliations:** 1 National Center for Electron Microscopy, Molecular Foundry, Lawrence Berkeley National Laboratory, 1 Cyclotron Road, Berkeley, California 94720, USA; 2 Department of Physics, University of Oregon, 1585 E 13th Avenue, Eugene, Oregon 97403, USA; 3 Gatan Inc., 5794 W Las Positas Boulevard, Pleasanton, California 94588, USA; 4 Department of Physics, Center for Electron Microscopy, Ulm University, Albert-Einstein-Allee 11, 89069 Ulm, Germany

## Abstract

The ability to image light elements in soft matter at atomic resolution enables unprecedented insight into the structure and properties of molecular heterostructures and beam-sensitive nanomaterials. In this study, we introduce a scanning transmission electron microscopy technique combining a pre-specimen phase plate designed to produce a probe with structured phase with a high-speed direct electron detector to generate nearly linear contrast images with high efficiency. We demonstrate this method by using both experiment and simulation to simultaneously image the atomic-scale structure of weakly scattering amorphous carbon and strongly scattering gold nanoparticles. Our method demonstrates strong contrast for both materials, making it a promising candidate for structural determination of heterogeneous soft/hard matter samples even at low electron doses comparable to traditional phase-contrast transmission electron microscopy. Simulated images demonstrate the extension of this technique to the challenging problem of structural determination of biological material at the surface of inorganic crystals.

## Abstract (web summary)

Scanning transmission electron microscopy is a powerful material probe, but constrained to large atomic number samples due to the issues of beam damage and weak scattering. Here, Ophus *et al.* propose a method that produces linear phase contrast in a focused electron beam to image dose-sensitive objects.

## Graphical abstract

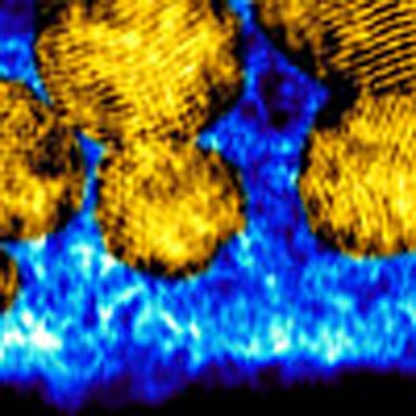

Structural analysis at atomic resolution is commonly used to provide deep insight into the functionality of structures in both biological and physical sciences. Recent examples include the enormous progress in dynamic structural biology^1^, direct imaging of screw dislocations via optical sectioning^2^, measurements of TiO_6_ octahedra in perovskite superlattices^3^ and many others. Transmission electron microscopy (TEM) and scanning TEM (STEM) are ubiquitous techniques for high-resolution analysis of both hard and soft matter structures due to the focusing capabilities of electron optics. The theoretical resolution limit for traditional TEM and STEM is 1–2 Å, which has further been extended to 0.5 Å with aberration correction^4^,^5^,^6^. This has significantly improved quantitative structural analysis in both TEM and STEM to sub-Å resolution and single-picometre precision in materials science where many materials can tolerate very high electron doses. Compared with STEM, TEM phase-contrast imaging is overwhelmingly preferred by the biological community because it provides an efficient means of imaging weak-phase objects at doses at or below 10 e^−^ Å^−2^. Resolution for biological materials is limited by the achievable signal-to-noise ratio (SNR) before the target structure is damaged or destroyed, rather than TEM information transfer^7^,^8^,^9^,^10^,^11^. The primary method currently used to solve the structure of dose-sensitive samples is single-particle reconstruction, from cryo-electron microscopy (cryo-EM). This method is very effective, but typically requires many thousands of identical particles isolated from each other so that the defocused signals from adjacent particles do not interfere. Further, information transfer depends on the defocus used, which has a large nonlinear effect on the contrast in the final image^12^,^13^,^14^,^15^. This effect is especially prevalent for phase-contrast high-resolution TEM (HRTEM) and requires careful inspection, numerical aberration correction and/or computer simulation for direct structural interpretation.

Image interpretation in STEM is typically simpler. The contrast and efficiency of STEM is controlled by the geometry of the post specimen, monolithic detectors which simply integrate over specific scattering angles in reciprocal space. The two most common STEM imaging techniques are annular dark field (ADF) that can produce incoherent image contrast roughly proportional to the projected mass thickness of the sample and bright field (BF), which can produce coherent image contrast similar to traditional TEM^15^,^16^. An alternative method called annular bright field is a new technique designed to directly image weakly scattering materials such as lithium and oxygen, but this imaging technique is difficult to optimize since the detector inner/outer angle ratio is set by the physical detector size^17^,^18^. ADF-STEM with incoherent image contrast is commonly used to produce interpretable images at atomic resolution based on high-angle scattering, but this process is relatively inefficient per incident electron, commonly requiring significantly more dose per unit area compared with TEM. This is especially limiting for light elements such as carbon, which scatter electrons very weakly. Thus, STEM is more commonly used for materials science and infrequently in biological sciences^19^,^20^.

Coherent, phase-contrast imaging in STEM is also possible using a differential measurement of the BF centre disk as initially proposed by Rose^21^. Dekkers and De Lang, Rose and Haider *et al.*^22^,^23^,^24^ also proposed using a STEM probe aberration corrector to form a probe containing a reference wave, which can be directly interpreted using a segmented detector. Ptychography is another dose-efficient phase-contrast method that utilizes full images of the transmitted electron diffraction pattern to reconstruct both the complex (real and imaginary) probe image and complex sample potential. Ptychography was demonstrated at atomic resolution for the first time by Nellist *et al.*^25^, with recent improvements in both computer algorithms and detectors^26^,^27^,^28^. One recent example of how phase-contrast imaging can be achieved in STEM is given by the work of Pennycook *et al.*^29^,^30^. Their method implements an elegant ptychographic reconstruction algorithm that uses subregions recorded on a pixel array detector to form efficient phase-contrast images.

STEM experiments can also be expanded using methods other than advanced detector geometries and computational algorithms. One example is the recent use of structured phase in electron microscopy, typically performed by placing a phase or amplitude plate in the probe-forming aperture to produce an electron probe with the desired properties. Diffraction gratings have been used in STEM to create vortex beams with orbital angular momentum^31^,^32^,^33^ and Bessel beams for very long depth-of-field imaging^34^.

Although all of these methods are important steps to improving STEM imaging beyond the simple mass-thickness contrast of ADF-STEM, it is highly desirable to develop a high-resolution imaging technique with directly interpretable contrast that can also operate with high efficiency to reduce beam damage. This could expand the use of STEM to solve important questions in the biological field, as well as hybrid hard/soft materials^35^,^36^.

In this manuscript, we present simulations and a proof-of-principle experiment for a new kind of phase-contrast electron microscopy called matched illumination and detector interferometry (MIDI)–STEM. MIDI–STEM combines the concepts of phase gratings, aberration correction, high-speed pixelated direct electron detectors and phase reconstruction using an interference pattern (such as in electron holography). The MIDI–STEM method produces almost ideal linear phase-contrast images over a wide range of spatial frequencies with very high efficiency and could potentially be used to image soft matter and beam-sensitive samples at atomic resolution.

## Results

### Description of MIDI–STEM experimental set-up

**Figure 1:**
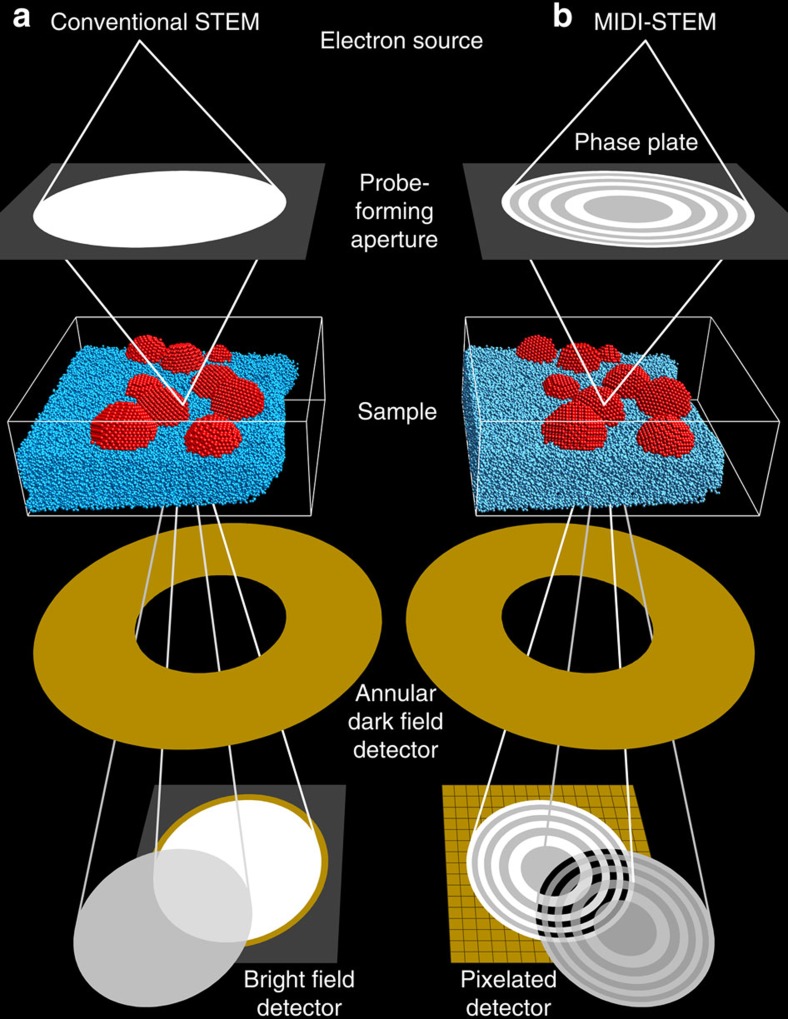
Experimental set-up for STEM experiments. (**a**) Conventional set-up, with a round probe-forming aperture and monolithic, single-pixel ADF and BF detectors below the sample. (**b**) MIDI–STEM set-up, with a patterned phase plate placed in the probe-forming aperture and a pixelated detector below the sample.

A simplified diagram comparing a conventional STEM (Fig. 1a) and MIDI–STEM (Fig. 1b) experimental set-up is shown in Fig. 1. In conventional STEM experiments, the probe is formed by a plane wave incident on a circular condenser aperture. Lens elements are used to create a circular electron beam with (approximately) constant phase, which converges to an atomic-scale probe at the sample plane. Electromagnetic deflectors scan the probe over the sample surface in a 2D grid pattern. As shown in Fig. 1a, post specimen, monolithic detectors integrate over regions of the scattered (dark field) or unscattered (bright field) electron diffraction pattern. Two common detector configurations are shown, an ADF detector and a BF detector.

In a MIDI–STEM experiment, diagrammed in Fig. 1b, a patterned phase plate is placed at the probe-forming aperture position. The phase plate consists of alternating concentric trenches with equal area where a thin SiN film has been patterned by a focused ion beam. Each alternating ring applies either a 0 or *π*/2-phase shift due to the local SiN thickness. This phase plate generates a probe with a built-in reference wave, which is then scanned across the sample as in traditional STEM imaging. In this case, the high-angle scattering signal is recorded by a traditional ADF detector, and a pixelated direct electron detector is used to record an image of the transmitted centre beam at each scanned position. The ADF detector produces a signal that is very similar to a conventional ADF-STEM experiment. The images of the transmitted centre beam are processed by fitting a virtual detector to match the geometry of the phase plate producing an approximately linear phase signal. The virtual detector consists of the even and odd-numbered annular rings formed by the phase plate, where the phase signal is given by the difference between the sum of all odd ring intensities minus the sum of all even ring intensities. Precise alignment of the virtual detector rings can be achieved using an image of the centre beam in vacuum or by averaging all diffraction pattern images and fitting ellipses to the ring edges^37^. The ability to match the virtual detector to the phase-plate geometry using post processing makes MIDI–STEM highly flexible to compensate for any errors in the phase plate itself or in the scanning electronics. It is also capable of utilizing almost any pre-specimen phase-plate design. Further technical details of the MIDI–STEM model are given in Supplementary Note 1 and Supplementary Fig. 1.

**Figure 2:**
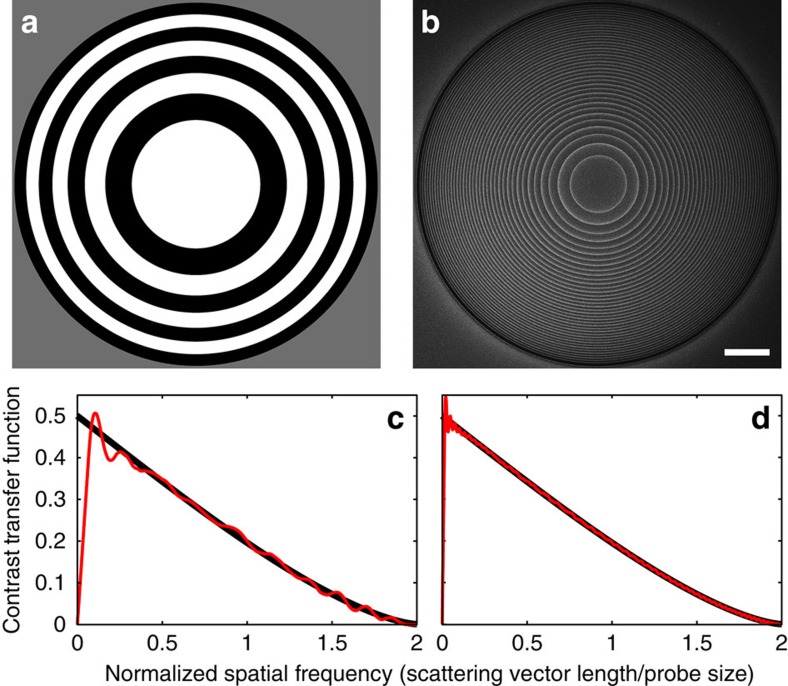
MIDI–STEM phase plates and resulting CTFs. (**a**) Schematic of a phase plate with 4 ring pairs, and (**b**) scanning electron microscopy image of the patterned phase plate with 20 ring pairs used in this study. Scale bar, 5 μm. The calculated CTFs for (**a**,**b**) are plotted in (**c**,**d**), respectively. Black diagonal lines show the CTF for an ideal phase-contrast STEM experiment.

The contrast transfer function (CTF) of a microscopy technique describes the measured contrast as a function of the scattering angle or spatial frequency^15^. A monotonically decreasing CTF that passes both low and high spatial frequencies is desirable for easy image interpretation. The CTF of MIDI–STEM can be calculated from the overlap region of the 0 and *π*/2 regions of the probe for the unscattered centre disk and a scattered disk. An example of a MIDI–STEM CTF is plotted in Fig. 2c, where the phase-plate geometry is shown in Fig. 2a. The scanning electron microscopy image in Fig. 2b shows the exact phase plate used in this study, which produces the CTF plotted in Fig. 2d. The geometric construction used to calculate MIDI–STEM CTFs is shown in more detail in Supplementary Figs 2, 3 and 4 and Supplementary Note 2.

### A MIDI–STEM experiment

**Figure 3:**
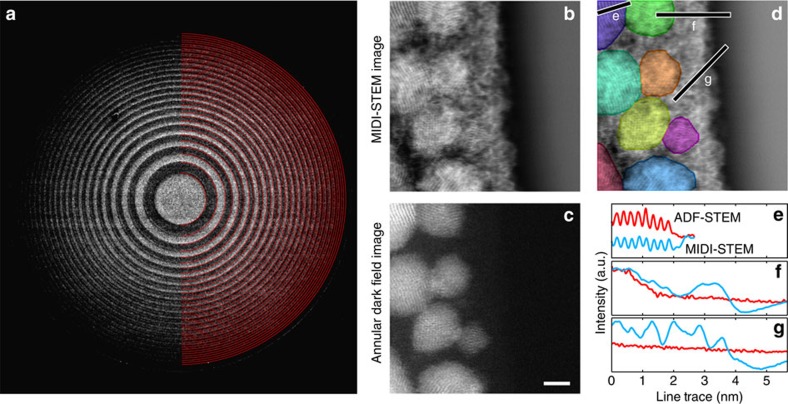
MIDI–STEM experiment of a heterogeneous sample. (**a**) The image of the centre beam averaged from all diffraction patterns. Simultaneously, recorded images of gold NPs on a thin carbon support (**b**) using a virtual detector (edges outlined by red lines) shown in (**a**,**c**) using a conventional ADF-STEM detector. Scale bar, 2 nm. (**d**) Same as (**b**), with gold NPs shaded with random colours to emphasize the surrounding carbon. (**e**–**g**) Line traces with positions shown in (**d**) for images (**b**,**c**).

We performed an MIDI–STEM experiment to image a highly heterogeneous sample consisting of randomly oriented cuboctahedral gold nanoparticles (NPs) supported on a thin amorphous carbon film to demonstrate the linear imaging capabilities of this technique. The average image of the centre beam from all probe positions is plotted in Fig. 3a, with the fitted edges of the virtual detector overlaid on the right half of the image as red lines. Note that the contrast has been scaled up to make the phase-plate rings visible. The ADF detector is not visible at this contrast level, but was positioned such that the inner detector angle was just beyond the edge of the outer-most phase-plate ring at 20 mrad.

In Fig. 3b,c, we show the MIDI–STEM image reconstructed using the matched virtual detector and the simultaneously recorded ADF detector image, respectively. The ADF image shows strong contrast for the gold NPs, and the atomic planes are visible in several NPs typically with the (111) plane spacing. The carbon support is very faintly visible in the ADF image, and though it can be distinguished from vacuum, no structural information can be obtained.

The MIDI–STEM image shown in Fig. 3b also shows good contrast for the NPs, with a similar SNR for the atomic planes as the ADF image in Fig. 3c. Furthermore, the MIDI–STEM image also shows very strong contrast for the carbon support, especially at the vacuum edge. The ADF image was used to color the area occupied by the gold NPs in Fig. 3d to emphasize the surrounding carbon structure. Inside the carbon film, we observe regions of correlated intensity between adjacent pixels in both the fast (horizontal) and slow (vertical) directions. Because each image pixel is a separate probe position representing a completely independent measurement, we ascribe these features to the atomic clustering characteristics of filament-like structures known to exist in amorphous carbon^38^. The gold NPs also have significant additional contrast that we interpret as amorphous carbon clustering around the particles. The source of this carbon could be from the sample fabrication process, from the surrounding substrate, or contamination from previous STEM scans used for focusing. This additional contrast is not visible in the ADF images (except perhaps as some weak ‘fuzziness') demonstrating that ADF imaging suppresses weakly scattering atoms such as carbon, which makes samples appear to be cleaner than they really are. ADF-STEM is essentially biased towards highly scattering materials, and this experiment demonstrates the capabilities of MIDI–STEM to simultaneously image low- and high-scattering materials.

Three line traces taken from the MIDI–STEM and ADF-STEM images of Fig. 3b,c are plotted in Fig. 3e–g. Figure 3e shows that both MIDI- and ADF-STEM are sensitive to the atomic lattice planes of the NPs, with approximately the same SNR. The left side of Fig. 3f shows that both methods show thickness contrast for an off-zone-axis NP, but the carbon substrate edge at the vacuum is essentially invisible in the ADF-STEM image, while strongly visible in the MIDI–STEM image. Finally, the trace in Fig. 3g along only amorphous carbon shows strong structural fluctuations in MIDI–STEM, and again no contrast in ADF-STEM. The MIDI–STEM image shows slowly varying intensity in the vacuum because it is a differential phase technique (similar to a high-pass filter) that cannot retrieve the d.c. component or very low spatial frequencies. This is evident in the CTF curves plotted in Fig. 2.

### Contrast transfer of MIDI-STEM images from simulation

**Figure 4:**
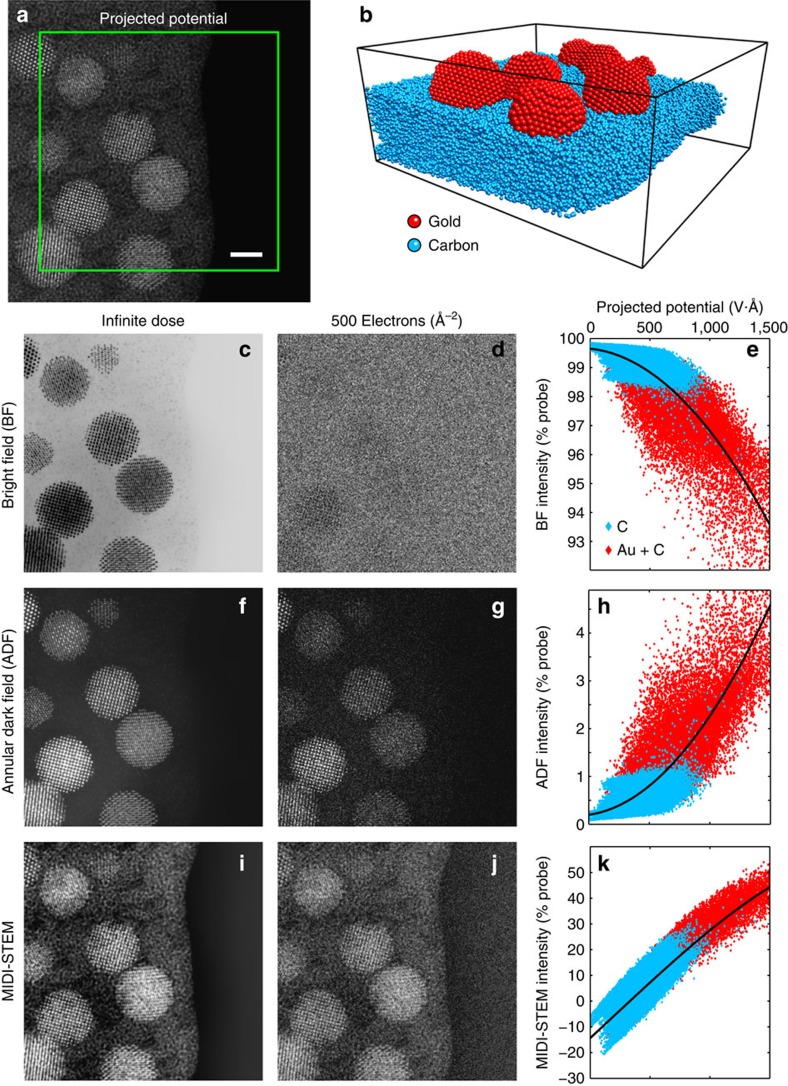
MIDI–STEM simulations of a heterogeneous sample. (**a**) The projected potential and (**b**) corresponding atomistic three-dimensional model. Scale bar, 2 nm. Images were generated from scanning over the area enclosed in the green box using two common STEM imaging modes, (**c**–**e**) BF-STEM and (**f**–**h**) ADF-STEM, and (**i**–**k**) MIDI–STEM. Simulations are shown for (**c**,**f**,**i**) infinite and (**d**,**g**,**j**) moderate dose. (**e**,**h**,**k**) Quantitative comparison between the projected potential and the measured infinite dose signal shows that MIDI–STEM is a significantly more linear measurement than traditional methods.

To validate our experimental results, we have simulated a MIDI–STEM experiment of a similar sample using the multislice method. The projected potential of the sample and atomistic model are shown in Fig. 4a,b. The simulated sample consists of randomly oriented cuboctahedral gold NPs attached to a wedge-shaped substrate of amorphous carbon. The realistic amorphous carbon atomic coordinates from^38^ were tiled into a wedge with a maximum thickness of 5 nm on the left side and a minimum thickness at the substrate/vacuum edge of 3 nm. No additional carbon was added on top of the gold NPs as seen in the experimental results. The simulated STEM scan of 300 × 300 probe positions with a probe position spacing of 0.5 Å was confined to the green box overlaid on the projected potential.

Three detector configurations are considered in Fig. 4c–k; a BF-STEM detector constructed by summing over the central probe disk from 0 to 17 mrad, a (low angle) ADF-STEM detector from 20 to 95 mrad and a MIDI–STEM virtual detector consisting of the electrons recorded on odd rings minus those recorded on even rings. Detector geometries were chosen to match the experimental set-up presented earlier although only experimental ADF- and MIDI–STEM signals were experimentally available. All simulated images are plotted using infinite dose (no noise) and with an intermediate dose of 500 e^−^Å^−2^. In addition, the images are quantitatively evaluated by plotting the measured signal intensity at infinite dose as a fraction of the total incident electrons versus the projected potential of the sample at each probe position. The pixels are separated into two groups corresponding to probe positions at only amorphous carbon (blue) or probe positions including both carbon and gold (red) in projection. A polynomial trend line (black) was fitted to all points as a guide for the eye. Note that at an accelerating voltage of 300 kV, a projected potential of 1,500 V Å roughly corresponds to a *π*-phase shift of the incident electron wave, and therefore this specimen does not obey the weak-phase approximation.

The BF- and ADF-STEM simulations are essentially complementary, as expected when using such a low inner angle for the ADF detector. Both show strong contrast for the gold NPs and weak contrast for the carbon substrate at infinite electron dose. However, when using a dose of only 500 e^−^Å^−2^, the BF-STEM signal is overwhelmed by noise; only faint outlines of the NPs are visible and the substrate is almost invisible. The ADF-STEM image produces relatively better contrast at lower dose, as the NPs show high contrast both for atomic columns and atomic planes. In this ADF-STEM image, the substrate can be differentiated from the vacuum, but no structural information can be obtained. A more typical high-angle ADF-STEM image with a large detector inner angle (>50 mrad) of this sample produces a slightly better image of the gold, but significantly less contrast for the carbon substrate.

The simulated MIDI–STEM image by comparison shows very strong contrast for both the NPs and the amorphous substrate. Even at an electron dose much lower than typical STEM experiments, atomic positions and planes are visible in the NPs, with contrast roughly equivalent to the ADF-STEM images. However, the contrast of the amorphous substrate has been significantly increased, and the atomic-scale details of the projected potential are visible in the infinite dose images. The finite dose MIDI–STEM image also shows many of the same structural details when comparing with the fine structure of the projected potential. Qualitatively, the efficiency of MIDI–STEM is explained by its ability to measure small scattering events due to the alternating rings of the phase plate. The plot of projected potential versus measured MIDI–STEM signal shows it is far more linear than BF- or ADF-STEM and has a much tighter distribution of measurements. Importantly, both the carbon and gold plus carbon signals fall on the same roughly linear curve. The primary sources of non-linearity in the MIDI–STEM measurement are the high-pass filtering effect of MIDI–STEM (a minor effect on the scale of a 20 nm field of view) and the decreased number of electrons available to scatter for thicker regions of the sample.

## Discussion

**Figure 5:**
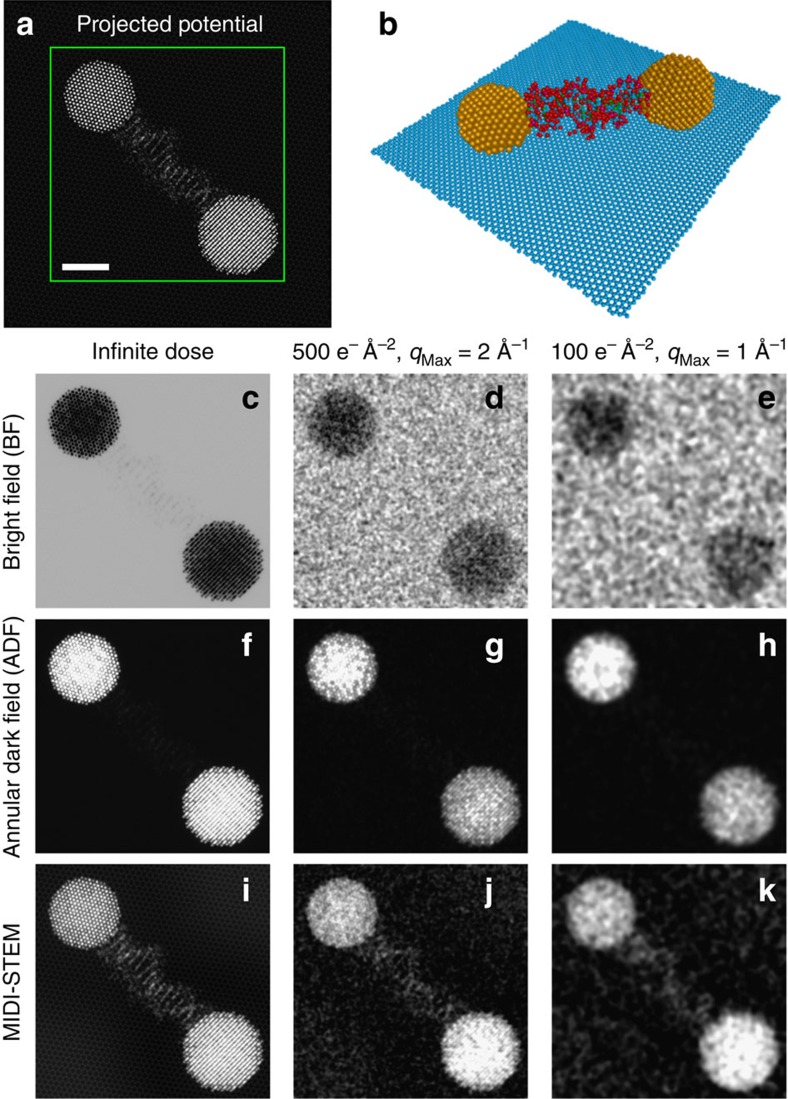
MIDI–STEM simulations of a biological–inorganic interface. (**a**) Projected potential of a DNA snippet connecting two gold NPs on a single layer of graphene substrate, and (**b**) corresponding atomistic three-dimensional model. Scale bar, 2 nm. Images were generated from scanning over the area enclosed in the green box using (**c**–**e**) BF-STEM, (**f**–**h**) ADF-STEM and (**i**–**k**) MIDI–STEM at infinite and two low electron doses. Conventional STEM probes were used for BF- and ADF-STEM, while 20 ring pairs were used for MIDI–STEM.

It is important to emphasize that the key strengths of this method are its applicability for beam-sensitive soft materials and the advantages of linear contrast transfer towards the study of hard/soft interfaces in materials science. We have reported both the experimental and theoretical validity of MIDI–STEM for a sample of gold NPs on an amorphous carbon support as a very general case of a highly heterogeneous sample as a proof of principle. To explore the limits of the MIDI–STEM method, we performed a multislice simulation of a DNA snippet connecting two gold NPs on a single layer of graphene, plotted in Fig. 5. DNA was chosen for its well-known structure with weak scattering and moderate dose sensitivity. The same microscope parameters and detectors as Fig. 4 were used, and electron doses of infinity, 500 and 100 e^−^Å^−2^ were simulated. However, unlike in Fig. 4c–h, in Fig. 5c–h the BF- and ADF-STEM simulations were performed using a conventional STEM probe without a phase plate. As above, neither the BF- nor ADF-STEM images show any appreciable contrast in the DNA section at a non-infinite electron dose. Conversely, the MIDI–STEM images (Fig. 5i–k) show linear contrast even at fairly low electron doses. Even at such low doses, MIDI–STEM produces enough contrast to identify not only the presence or absence of a bio-molecule, but also the shape envelope and orientation, while being in focus. MIDI–STEM is therefore a promising technique for imaging relatively radiation hard bio-molecules and heterostructures such as hard/soft interfaces. For the most dose-sensitive bio-molecules, highly defocused cryo-EM is a more efficient imaging method, but many samples cannot meet the cryo-EM requirements of many well-separated identical structures, with no strongly scattering components. The same atomic coordinates are used for a comparison with phase contrast, defocused HRTEM imaging in Supplementary Note 3 and Supplementary Fig. 5, using the deconvolution methods described in ref. 14. These simulations show that more information can be recovered from HRTEM imaging, but this requires large defocus values that can produce delocalization artifacts.

In summary, we have experimentally demonstrated the MIDI–STEM imaging method with great promise for improving the contrast in STEM images for weakly scattering materials. We also performed multislice simulations of a sample realistically modelled after our experiment to confirm our interpretation of the experimental results. In this experiment, we imaged gold NPs on an amorphous carbon support, using a pixelated direct electron detector to construct the virtual detectors necessary for MIDI–STEM while simultaneously recording an ADF-STEM image. The MIDI–STEM image simultaneously showed atomic-plane contrast for highly scattering gold NPs and the amorphous structure of carbon regions. Structural features on the near-atomic scale were clearly visible in the amorphous carbon film, showing that MIDI–STEM is a promising candidate to directly image samples consisting of both hard and soft matter at atomic or near-atomic resolution using relatively low electron doses. The primary advantages of MIDI–STEM are high signal efficiency, good transfer of low spatial frequency information and the ability to image while in focus to minimize signal delocalization. MIDI–STEM should also allow the possibility of post-acquisition software aberration correction using pychographic methods.

## Methods

### Experimental

All experimental results presented in this paper were recorded on TEAM I, an aberration-corrected FEI Titan 80–300 operated in STEM mode at 300 kV with a convergence semi-angle of 17.2 mrad. The phase-plate geometry used to form the MIDI–STEM probes were equal-area Fresnel zone plates with 20 ring pairs, fabricated using focused ion beam milling of a SiN membrane^33^. The transmitted electron diffraction pattern at each probe position was recorded using a Gatan K2 IS direct electron detector with 3,840 × 3,712 pixels, operated at 400 frames per second and binned by 2. The camera acquisition and probe scanning were synchronized using a Gatan Digiscan. The probe was scanned over the 14.5 nm field of view with 256 × 256 probe positions to create a 256 × 256 × 1,920 × 1,792 four-dimensional STEM data set consisting of 420 GB of raw images.

### Analysis and Simulation

Post processing to fit the virtual detector was done using custom scripts in MATLAB. All multislice simulations were performed using custom MATLAB codes that follows the methods of Kirkland^15^, using the same microscope parameters as in the experiment and eight frozen phonon configurations. STEM probes were spaced by 0.5 Å, and the simulation pixel size was 0.2 Å. Experimental and simulated microscope parameters were optimized to give the highest contrast. A model detailing the geometric CTF calculations is given in the Supplementary Notes 1 and 2.

## Additional information

**How to cite this article:** Ophus, C. *et al.* Efficient linear phase contrast in scanning transmission electron microscopy with matched illumination and detector interferometry. *Nat. Commun.* 7:10719 doi: 10.1038/ncomms10719 (2016).

## Supplementary Material

Supplementary Information Supplementary Figures 1-5, Supplementary Notes 1-3 and Supplementary References
