# Supplementary material for: Efficient linear phase contrast in scanning transmission electron microscopy with matched illumination and detector interferometry
Source: Nat Commun. 2016 Feb 29;7:10719. doi: 10.1038/ncomms10719 (PMC4773450; doi:10.1038/ncomms10719)
Supplement: Supplementary Information — Supplementary Figures 1-5, Supplementary Notes 1-3 and Supplementary References [file ncomms10719-s1.pdf]

Supplementary Figures

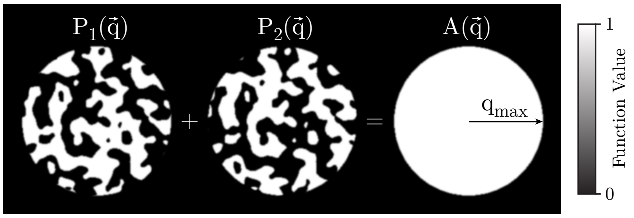

Supplementary Figure 1: Example non-overlapping, binary probe functions $P_1(\vec{q})$ and $P_2(\vec{q})$, that add to form a top hat function $A(\vec{q})$.

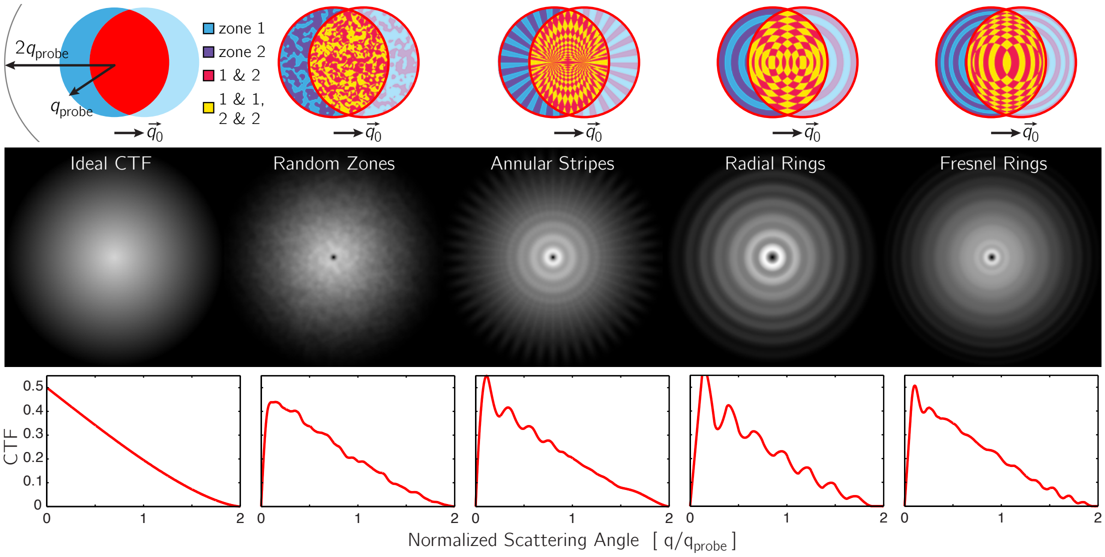

Supplementary Figure 2: (top) Various MIDI-STEM probes, (center) the associated contrast transfer functions, (bottom) and radially-averaged CTFs. Zone 1 refers to 0 rad phase shift and zone 2 to the region with an induced $\pi/2$ rads phase shift.

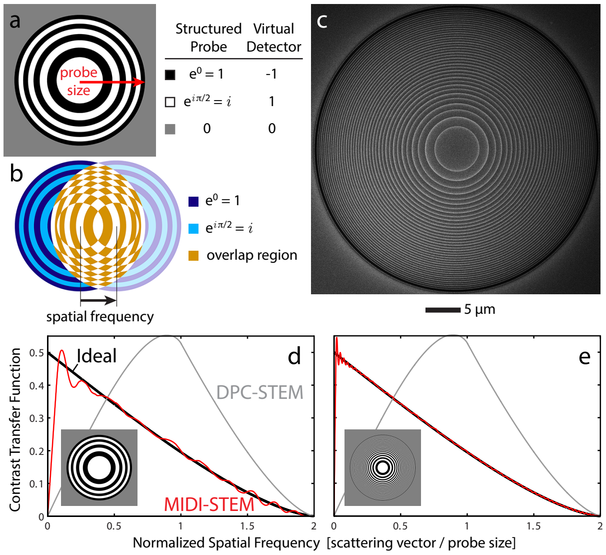

Supplementary Figure 3: (a) Schematic of a patterned phase plate with 4 ring pairs, showing regions of the probe with a 0 rad phase shift, a pi/2 rad phase shift, and zero intensity. The matching virtual detector will have the same geometry, with the weighting matching the probe regions equal to -1, 1, and 0 respectively. (b) A diagram showing the overlapping signal given by an unscattered MIDI-STEM probe disk (brighter colors) and a scattered probe disk. The yellow regions mark where either a 0 rad region overlaps with a pi/2 rad region, or vice versa. The ideal CTF is given by this total overlapping area for a given spatial frequency. (c) Scanning electron microscopy (SEM) image of a patterned phase plate with 20 ring pairs that was used in this study. The calculated CTFs for (a) and (c) are plotted in (d) and (e) respectively. The associated MIDI-STEM probes are inset.

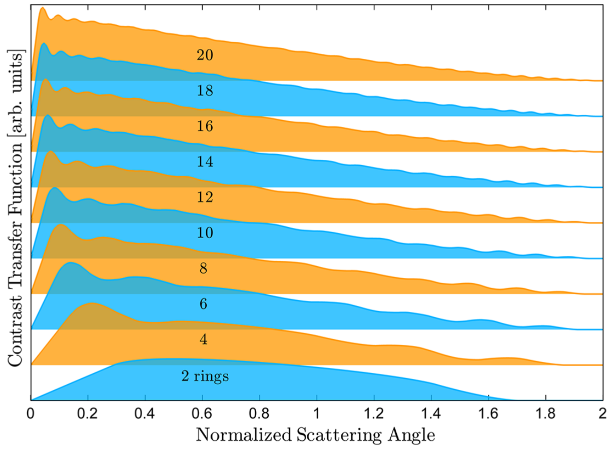

Supplementary Figure 4: Dependence of the contrast transfer function for MIDI-STEM probes with Fresnel geometry on the number of rings (equal number of 0 and $\pi/2$ phase shift zones.).

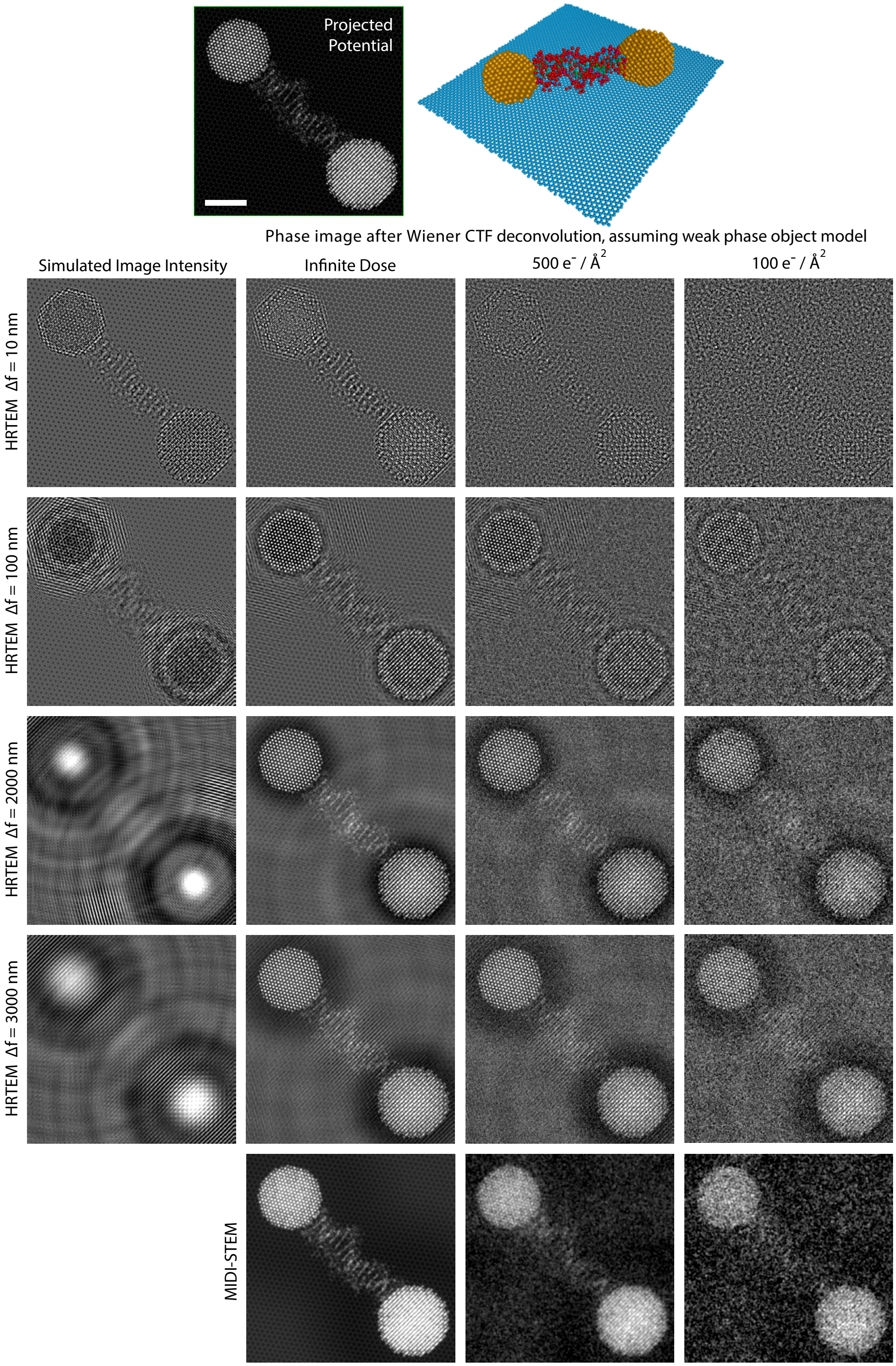

Supplementary Figure 5: Multislice simulations of MIDI-STEM imaging of a DNA snippet connecting two gold NPs on a single layer of graphene substrate. The projected potential and atomistic 3D model are shown at the top, with a scale bar of 2 nm. HRTEM images recorded at infinite and two low electron doses are shown below. To the right of these, reconstructed phase images assuming an ideal CTF and the weak-phase object model are shown. At the bottom, a MIDI-STEM simulation from 20 ring pairs is shown for comparison, also with Wiener deconvolution.

Supplementary Notes

Supplementary Note 1. MIDI-STEM Contrast Model

We will now derive a model for contrast in MIDI-STEM. Following a method similar to Kirkland [1], we start by defining a initial real space probe function $\psi_0(\vec{r})$ and a transmission function $t(\vec{r})$ that represents the sample potential over all real space coordinates $\vec{r}$. The evolution of $\psi_0(\vec{r})$ after interaction with an infinitely thin sample is given by

$$\psi(\vec{r}) = \psi_0(\vec{r})t(\vec{r}), \tag{1}$$

where $\psi(\vec{r})$ represents the electron probe after interacting with the sample. If the specimen is a weak phase object, it can be described by a scalar field $\phi(\vec{r})$ where $|\phi(\vec{r})| \ll 1$ everywhere, and the transmission function is equal to

$$t(\vec{r}) = 1 + i\phi(\vec{r}). \tag{2}$$

Combining Eqs. 1 and 2 and taking the 2D Fourier transform $\mathcal{F}\{\,\}$ of both sides yields

$$\begin{aligned} \mathcal{F}\{\psi(\vec{r})\} &= \mathcal{F}\{\psi_0(\vec{r})\,[1 + i\phi(\vec{r})]\}, \\ \Psi(\vec{q}) &= \Psi_0(\vec{q}) \otimes [\delta(\vec{q}) + i\Phi(\vec{q})]\,, \end{aligned} \tag{3}$$

where $\Psi(\vec{q})$, $\Psi_0(\vec{q})$ and $\Phi(\vec{q})$ are the Fourier transforms over the 2D diffraction space coordinates $\vec{r}$ for $\psi(\vec{r})$, $\psi_0(\vec{r})$ and $\phi(\vec{r})$ respectively, and $\otimes$ represents the two-dimensional convolution operator

$$f(\vec{q}) \otimes h(\vec{q}) = \iint_{\vec{k}} f(\vec{k})h(\vec{q} - \vec{k})d\vec{k}. \tag{4}$$

Next we define a probe shape in terms of two zones, $P_1(\vec{q})$ and $P_2(\vec{q})$. These two functions are defined to be non-overlapping binary functions, with each diffraction space pixel equal to zero or one. The shape of these functions does not matter, as long as they have equal total area, and they are defined to add up to a top hat function $A(\vec{q})$, i.e.

$$\begin{aligned} A(\vec{q}) &= P_1(\vec{q}) + P_2(\vec{q}), \\ A(\vec{q}) &= \begin{cases} 1 & \text{where } q \leq q_{\max} \\ 0 & \text{where } q > q_{\max}, \end{cases} \end{aligned} \tag{5}$$

where $q_{\max}$ is the maximum scattering angle set by the probe-forming aperture. An example of two zones that meet these constraints is plotted in Supplementary Figure 1. As in the previous paragraph, we will define a MIDI-STEM probe $\Psi_0(\vec{q})$ in Fourier space by using a binary phase plate with phase shifts of 0 and $\pi/2$ corresponding to the zones $P_1(\vec{q})$ and $P_2(\vec{q})$ respectively, giving

$$\Psi_0(\vec{q}) = P_1(\vec{q}) + iP_2(\vec{q}). \tag{6}$$

Using this probe function in Eq. 3 yields

$$\begin{aligned} \Psi(\vec{q}) &= [P_1(\vec{q}) + iP_2(\vec{q})] \otimes [\delta(\vec{q}) + i\Phi(\vec{q})] \\ &= [P_1(\vec{q}) - P_2(\vec{q}) \otimes \Phi(\vec{q})] + i\,[P_2(\vec{q}) + P_1(\vec{q}) \otimes \Phi(\vec{q})] \end{aligned} \tag{7}$$

The measured intensity at the detector plane will be equal to the modulus squared of the probe wavefunction, $|\Psi(\vec{q})|^2$. Computing it for the above expression and keeping only the linear $\Phi(\vec{q})$ terms because $|\Phi(\vec{q})| \ll 1$ gives

$$|\Psi(\vec{q})|^2 = {P_1}^2(\vec{q}) + {P_2}^2(\vec{q}) - 2P_1(\vec{q})\,[P_2(\vec{q}) \otimes \Phi(\vec{q})] + 2P_2(\vec{q})\,[P_1(\vec{q}) \otimes \Phi(\vec{q})]$$

As the name "matched illumination and detector interferometry" implies, the virtual detector geometry should be matched to the geometry of the phase plate (i.e. to the above contrast equation of Eq. 8). Therefore we define the detector operator $\mathcal{D}\{\,\}$ for MIDI-STEM to be the difference between the summed intensity of electrons that land in the part of the probe defined by $P_1(\vec{q})$ and the electrons that land in the $P_2(\vec{q})$ zone,

$$\mathcal{D}\{|\Psi(\vec{q})|^2\} = \iint_{\vec{q}} |\Psi(\vec{q})|^2\,[P_2(\vec{q}) - P_1(\vec{q})]\,d\vec{q} \tag{8}$$

Applying this detector operator to the first two terms of Eq. 8 gives

$$\begin{aligned} &= \iint\limits_{\vec{q}} \left[{P_1}^2(\vec{q}) + {P_2}^2(\vec{q})\right] \left[P_2(\vec{q}) - P_1(\vec{q})\right] d\vec{q} \\ &= \iint\limits_{\vec{q}} A(\vec{q}) \left[P_2(\vec{q}) - P_1(\vec{q})\right] d\vec{q} = 0, \end{aligned} \tag{9}$$

because ${P_1}^2(\vec{q}) = P_1(\vec{q})$, ${P_2}^2(\vec{q}) = P_2(\vec{q})$, and the non-zero areas of $P_1(\vec{q})$ and $P_2(\vec{q})$ were defined to be equal. Using the commutative property of convolution, the identity $P_1(\vec{q})P_2(\vec{q}) = 0$, and applying the detector operator to the third and fourth terms of Eq. 8 yields

$$\begin{aligned} &\mathcal{D}\left\{|\Psi(\vec{q})|^2\right\} \\ &= 2 \iint\limits_{\vec{q}} P_1(\vec{q}) d\vec{q} \iint\limits_{\vec{k}} P_2(\vec{k}) \Phi(\vec{q} - \vec{k}) d\vec{k} + 2 \iint\limits_{\vec{q}} P_2(\vec{q}) d\vec{q} \iint\limits_{\vec{k}} P_1(\vec{k}) \Phi(\vec{q} - \vec{k}) d\vec{k} \\ &= 2 \iint\limits_{\vec{q}} \left[P_1(\vec{q}) \otimes P_2(-\vec{q}) + P_2(\vec{q}) \otimes P_1(-\vec{q})\right] \Phi(\vec{q})\, d\vec{q} \end{aligned} \tag{10}$$

If $P_1(\vec{q})$ and $P_2(\vec{q})$ are even (radially symmetric), then

$$\mathcal{D}\{|\Psi(\vec{q})|^2\} = 4 \iint\limits_{\vec{q}} \left[P_1(\vec{q}) \otimes P_2(\vec{q})\right] \Phi(\vec{q}) d\vec{q}. \tag{11}$$

We now see that the combination of this structured phase with a matched, differential detector produces a linear phase measurement of the weak-phase signal of the object wave, multiplied by a contrast transfer function (CTF) given by $H(\vec{q}) = 4\, P_1(\vec{q}) \otimes P_2(\vec{q})$. We will explore MIDI-STEM CTF functions in detail in the next section. We can measure the sample phase at a position $\vec{r}_0$ in the sample plane (in real space) by multiplying Eq. 11 by the Fourier shift operator $\exp(2\pi i \vec{q} \cdot \vec{r}_0)$, defining a measurement operator

$$m(\vec{r}_0) = \iint\limits_{\vec{q}} H(\vec{q}) \Phi(\vec{q}) \exp(2\pi i \vec{q} \cdot \vec{r}_0) d\vec{q}. \tag{12}$$

Noticing that this expression is equivalent to an inverse Fourier transform $\mathcal{F}^{-1}\{\}$ with respect to $\vec{q}$, we can write

$$m(\vec{r}_0) = h(\vec{r}_0) \otimes \phi(\vec{r}_0), \tag{13}$$

where $h(\vec{r})$ is the inverse Fourier transform of $H(\vec{q})$, also known as the point spread function (PSF). The measured MIDI-STEM signal is therefore a linear measurement at position $\vec{r}_0$ of the object's phase shift $\phi(\vec{r}_0)$, convolved with a PSF of

$$h(\vec{r}) = 4\, p_1(\vec{r})\, p_2(\vec{r}), \tag{14}$$

where $p_1(\vec{r})$ and $p_2(\vec{r})$ are the inverse Fourier transforms of $P_1(\vec{q})$ and $P_2(\vec{q})$ respectively. Note that if $P_1(\vec{q})$ and $P_2(\vec{q})$ are not even (not radially symmetric), then the full expression given in Eq. 10 must be used. Then, the CTF will be defined as

$$H(\vec{q}) = 2 \left[P_1(\vec{q}) \otimes P_2(-\vec{q}) + P_2(\vec{q}) \otimes P_1(-\vec{q})\right], \tag{15}$$

and the PSF will be equal to

$$h(\vec{r}) = 2 \left[p_1(\vec{r}) p_2(-\vec{r}) + p_2(\vec{r}) p_1(-\vec{r})\right]. \tag{16}$$

Supplementary Note 2. Contrast Transfer Functions for MIDI-STEM

The CTF refers to how contrast varies in a phase measurement for a weak phase object as a function of the scattering vector q_0. In MIDI-STEM, the CTF is essentially defined by the geometric overlap of zones 1 and 2 (and vice versa) when a copy of the probe mask is moved by an amount corresponding to q_0. Examples of this procedure are plotted in Supplementary Figure 2 for several phase plate geometries. The first example in Supplementary Figure 2 shows the CTF for an ideal probe, for an idealized experiment. This CTF would image the phase in a perfectly incoherent manner with a maximum scattering angle of $2\,q_{\mathrm{probe}}$. Such an ideal probe would be impossible to construct with current equipment, but it shows the ultimate limit of the technique.

The MIDI-STEM method will produce a CTF very close to the ideal CTF as long as the two zones have roughly the same area, and contain a mixture of high and low spatial frequencies. A randomly generated phase plate and its CTF are shown as the second example geometry in Supplementary Figure 2. The primary difference from the ideal CTF is that the constant DC phase component, at the center of the CTF, cannot be measured. Generating a linear phase image relies on knowing the phase shift pattern added to the probe with high accuracy. For this reason, a randomly generated phase plate would be a poor choice, since any mismatch between the virtual detector and structured illumination will cause a loss of information. Additionally, since the electron probe cannot be made perfectly coherent and all detectors have an incoherent point spread function (PSF), small features in the electron probe formed at the sample are undesirable.

Two more examples plotted in Supplementary Figure 2 show equally spaced annular stripes and equal width radial rings. These probe types produce CTFs with undesirable annular streaking and radial ringing, respectively. Therefore, we want a probe design with radial symmetry, and as few repeated spacings as possible. One such design is a Fresnel zone plate. In this geometry, each ring has the same area, meaning the ring radii vary with the square root of their radii. The CTF asymptotically approaches the ideal CTF as the number of rings is increased. The total number of rings will be limited by the minimum ring width measurable on the pixelated detector and by the coherence of the microscope. Supplementary Figure 3 shows how the CTF for MIDI-STEM was calculated geometrically for our experimental phase gratings.

To clearly show how increasing the number of Fresnel zones (shrinking the feature sizes of the structured probe) affects the CTF, we have plotted CTFs for 2 to 20 rings in Supplementary Figure 4. As the number of rings is increased, the CTF asymptotically approaches the CTF for the ideal probe plotted in Supplementary Figure 2. Both the highest and lowest spatial frequency zero crossings improve, approaching normalized scattering angles of 0 and 2 respectively. The primary limit for increasing the number of rings is that the detector point spread function will eventually smear out the signal of adjacent rings, causing a loss of signal.

Supplementary Note 3. Simulations Comparing MIDI-STEM to HRTEM

An extended version of Fig. 5 in the manuscript is plotted in Supplementary Figure 5. The top two panels show the projected potential and atomic structure for a short segment of DNA linking two gold nanoparticles. Multislice simulations for high resolution transmission electron microscopy (HRTEM) were performed using an ideal microscope (i.e. no aberrations or coherence envelopes were used, other than thermal motion of the atoms). The leftmost column of Supplementary Figure 5 shows HRTEM images (the amplitude of the exit wave) at different defocus values. The next 3 columns show reconstructions of the phase, which were calculated using a Wiener filter deconvolution of the ideal CTF due to defocus [2]. Three examples are shown for each defocus: infinite dose, 500 eV/Å^2, and 100 eV/Å^2.

At small defocus values, the simulated HRTEM amplitude images and reconstructed phase images show very little contrast using finite doses. HRTEM imaging with CTF-deconvolution performs well for large defocus values, on the scale of single-digit micrometers. However in these images, signal from the gold nanoparticles is highly delocalized (as it is a strong phase object), and the contrast at the interface between gold and the DNA snippet is obscured. The defocus value of 3000 nm shows that the delocalized contrast from a gold nanoparticle can easily generate additional features (in this case lattice planes) overlaid on top of the DNA structure.

By comparison, the MIDI-STEM images (bottom row) are generated while in-focus. This prevents the delocalization of the hard-matter particles from obscuring the soft-matter portion of the sample. We note however that the signal transfer of HRTEM is approximately double that of MIDI-STEM. This can be seen by the fact that the graphene lattice is more visible in the HRTEM images than the MIDI-STEM images, at non-infinite doses. Thus for samples consisting only of weak phase objects, we expect HRTEM to be the better technique. Note that Wiener filter deconvolution was also used for the MIDI-STEM images here, for a better comparison to the standard HRTEM imaging method.

Supplementary References

[1] EJ Kirkland, *Advanced computing in electron microscopy* (Springer Science & Business Media, 2010).

[2] KH Downing and RM Glaeser, "Restoration of weak phase-contrast images recorded with a high degree of defocus: the twin image problem associated with CTF correction," Ultramic. **108**, 921–928 (2008).
